# Impact of resveratrol-loaded liposomal nanocarriers on heat-stressed broiler chickens: Effects on performance, sirtuin expression, oxidative stress regulators, and muscle building factors

**DOI:** 10.3389/fvets.2023.1137896

**Published:** 2023-03-28

**Authors:** Asmaa T. Y. Kishawy, Doaa Ibrahim, Elshimaa M. Roushdy, Amira Moustafa, Fatma Eldemery, Elham M. Hussein, Fardos A. M. Hassan, Sara T. Elazab, Mohamed Tharwat Elabbasy, Raheela Kanwal, Walid M. Kamel, Mohamed R. Atteya, Asmaa W. Zaglool

**Affiliations:** ^1^Department of Nutrition and Clinical Nutrition, Faculty of Veterinary Medicine, Zagazig University, Zagazig, Egypt; ^2^Department of Animal Wealth Development, Animal Breeding, and Production, Faculty of Veterinary Medicine, Zagazig University, Zagazig, Egypt; ^3^Department of Physiology, Faculty of Veterinary Medicine, Zagazig University, Zagazig, Egypt; ^4^Department of Hygiene and Zoonoses, Faculty of Veterinary Medicine, Mansoura University, Mansoura, Egypt; ^5^Physics Department, Faculty of Science, Zagazig University, Zagazig, Egypt; ^6^Department of Animal Wealth Development, Veterinary Economics, and Farm Management, Faculty of Veterinary Medicine, Zagazig University, Zagazig, Egypt; ^7^Department of Pharmacology, Faculty of Veterinary Medicine, Mansoura University, Mansoura, Egypt; ^8^Department of Public Health, College of Public Health and Health Informatics, Ha'il University, Ha'il, Saudi Arabia; ^9^Food Control Department, Faculty of Veterinary Medicine, Zagazig University, Zagazig, Egypt; ^10^Department of Physical Therapy, College of Applied Medical Sciences, University of Ha'il, Ha'il, Saudi Arabia; ^11^Department of Public Health, College of Public Health and Health Informatics, University of Hail, Ha'il, Saudi Arabia; ^12^Department of Animal Wealth Development, Genetic, and Genetic Engineering, Faculty of Veterinary Medicine, Zagazig University, Zagazig, Egypt

**Keywords:** resveratrol-loaded liposome, broiler, performance, myogenic factor, sirtuin

## Abstract

Climate change is considered to be the primary cause of heat stress (HS) in broiler chickens. Owing to the unique properties of extracted polyphenols, resveratrol-loaded liposomal nanoparticles (Resv-Lipo NPs) were first explored to mitigate the harmful effects of HS. The dietary role of Resv-Lipo NPs in heat-stressed birds was investigated based on their growth performance, antioxidative potential, and the expression of heat shock proteins, sirtuins, antioxidant, immune, and muscle-building related genes. A total of 250 1-day-old Ross 308 broiler chickens were divided into five experimental groups (5 replicates/group, 10 birds/replicate) for 42 days as follows: the control group was fed a basal diet and reared in thermoneutral conditions, and the other four HS groups were fed a basal diet supplemented with Resv-Lipo NPsI, II, and III at the levels of 0, 50, 100, and 150 mg/kg diet, respectively. The results indicated that supplementation with Resv-Lipo NP improved the growth rate of the HS group. The Resv-Lipo NP group showed the most significant improvement in body weight gain (*p* < 0.05) and FCR. Additionally, post-HS exposure, the groups that received Resv-Lipo NPs showed restored functions of the kidney and the liver as well as improvements in the lipid profile. The restoration occurred especially at higher levels in the Resv-Lipo NP group compared to the HS group. The elevated corticosterone and T3 and T4 hormone levels in the HS group returned to the normal range in the Resv-Lipo NPsIII group. Additionally, the HS groups supplemented with Resv-Lipo NPs showed an improvement in serum and muscle antioxidant biomarkers. The upregulation of the muscle and intestinal antioxidant-related genes (*SOD, CAT, GSH-PX, NR-f2*, and *HO-1*) and the muscle-building genes (myostatin, *Myo*D, and *mTOR*) was observed with increasing the level of Resv-Lipo NPs. Heat stress upregulated heat shock proteins (HSP) 70 and 90 gene expression, which was restored to normal levels in HS+Resv-Lipo NPsIII. Moreover, the expression of sirtuin 1, 3, and 7 (*SIRT1, SIRT3, and SIRT7*) genes was increased (*p* < 0.05) in the liver of the HS groups that received Resv-Lipo NPs in a dose-dependent manner. Notably, the upregulation of proinflammatory cytokines in the HS group was restored in the HS groups that received Resv-Lipo NPs. Supplementation with Resv-Lipo NPs can mitigate the harmful impact of HS and consequently improve the performance of broiler chickens.

## 1. Introduction

High temperatures are one of the most lethal stressors in the poultry industry. Currently, the change in climate due to global warming is considered the main cause of heat stress (HS) facing poultry farming, especially broiler chickens, compared with other domestic animals (1). The most common complications that have been associated with exposure to high environmental temperatures in broiler chickens are the reduction in growth performance and survival rate, as well as metabolic disorders, immune suppression, and meat quality deterioration (2–4). These concerns were mainly caused by oxidative injury due to HS, which created a redox imbalance (5). The generation of free radicals involving reactive oxygen species (ROS) raised from oxidative stress can affect the body cells and cause damage to all biological molecules such as DNA, proteins, and fats, leading to cellular injury and pathological complications (6). Among the key events associated with HS promotion is the excessive expression of heat shock proteins (HSP), which are cytoprotective proteins that improve the tolerance and survival rates of stressed cells (5). The supplementation of bioactive natural antioxidants has recently been discovered as a promising approach to combat the deleterious impacts of free radicals generated by heat stress (7, 8). Resveratrol (RESV) is a natural bioactive polyphenol with a strong antioxidant capacity and anti-inflammatory and anti-aging impacts (9, 10). Resveratrol impedes glutathione disulfide formation and maintains glutathione in a reduced state, thus protecting cells from harmful reactions caused by free radicals (11). Moreover, resveratrol was shown to protect DNA from oxidative injury (12). Resveratrol is a known activator of the sirtuin family (especially sirtuin 1—*SIRT1*) (13, 14). Sirtuins can protect organisms from oxidative stress associated with cellular damage, stimulate DNA permanence, and reduce several age-related dysfunctions, such as metabolic abnormalities, neurodegeneration, and cancer (15, 16). Special attention has been given to the impact of resveratrol as a functional feed, which mitigates the heat-stress-reduced antioxidant functions in broiler chickens (10, 17, 18). Nevertheless, similar to several polyphenolic compounds, RESV is characterized by poor bioavailability, inadequate absorption, and rapid metabolism following oral administration. Therefore, higher doses of RESV are needed to achieve its significant favorable impacts (19). The incorporation of RESV into lipid nanosystems as liposomes has an inhibitory impact on lipid peroxidation, COX activity, and NO production, indicating its anti-inflammatory and antioxidant effects (20). RESV-loaded nanoparticles have been reported to protect cells against oxidative stress mediators by reducing ROS production (21). Encapsulation of phenolic compounds with the aid of liposomes is critical for their chemical and biological protection (22). Liposomes are considered biocompatible carriers that can be made from lipids with physicochemical functions and loaded with compounds of various lipophilic and hydrophilic natures (23, 24). Specifically, lipophilic ingredients, such as RESV, are usually integrated into the limiting lipid bilayer (25). Liposomes also allow the prolonged release of RESV and increase its stability. Furthermore, it has been demonstrated that loading with liposomes is an efficient way to prolong the release, increase the stability of RESV, and protect it from light and other deteriorative processes (26). The bioactive compounds that are incorporated into liposomes can be protected from degradation, which, in turn, improves their stability and solubility (27). Loading of resveratrol into liposomes nano carriers was shown to be effective in cell-stress response (28). Thus, we hypothesized that loading RESV in liposomes might enhance its functional properties and, subsequently, promote the productive performance of broiler chickens. To the best of our knowledge, there have been no studies on the role of RESV-loaded liposomal nanocarriers on gene expression modulation in heat-stressed broiler chickens. Taking all of this into consideration, the current study was conducted to bridge the previous knowledge gap by evaluating the influence of RESV-loaded liposomal nanoparticles on growth, serum antioxidant, and immune-related parameters together with their promising role on the expression of heat shock proteins, sirtuin family members, and myogenic regulatory factors.

## 2. Materials and methods

### 2.1. Birds' management and housing systems

In this study, two housing systems were applied in a controlled environment. The first system was the thermoneutral housing system, in which birds were reared from 1 to 42 days under standard brooding practice with a starting temperature of 32°C that decreased gradually by 2°C each week until it reached 20±1°C in the 6th week. The second system was the high-temperature housing system (heat-stressed system; HS), in which birds were reared from 1 to 42 days under a controlled high environmental temperature (birds were exposed to heat stress for 6 h per day at a temperature of 36 ± 2 °C). Both housing systems were supplemented with thermostatic electric heaters, electric fans for air circulation, and suction fans. The birds in both rearing systems were reared on the floor with sawdust litter in pins at a density of 10 birds/m^2^. Each pin represented a replica that was separated from each other by wire mesh and supplied with a manual filling feeder and drinker. Continuous lighting was established, and the relative humidity ranged from 35 to 50% throughout the experiment.

### 2.2. Diets and experimental design

A local hatchery obtained two hundred and fifty 1-day-old male chicks of a commercial meat type (Ross308). On arrival, they were weighed and randomly divided into five equal experimental groups. Each group contained five replicates with ten chicks each. The basal diets were formulated according to the nutrition specification for Ross308-Broiler (29). The diets were divided into three stages: The initial stage, known as the starter ration, lasted for the first 10 days. This was followed by the grower ration, which was fed for 24 days. Finally, the finisher ration was introduced and fed from day 25 to day 45 (Table 1).

**Table 1:** Ingredients and chemical composition percentage of the control diet.

**Ingredients**	**Experimental diets**		
	**Starter**	**Grower**	**Finisher**
Yellow corn	56	60.6	62
Soybean meal, 48%	34.86	29	25
Corn gluten, 60%	3.5	4.5	4
Wheat bran	-	-	1.9
Soybean oil	1.8	2	3.66
Calcium carbonate	1	1	0.9
Calcium dibasic phosphate	1.8	1.9	1.6
Common salt	0.3	0.3	0.3
Premix^*^	0.3	0.3	0.3
DL-Methionine, 98%	0.18	0.14	0.11
Lysine, Hcl, 78%	0.16	0.16	0.13
Anti-mycotoxin	0.1	0.1	0.1
**Calculated composition**			
Metabolize energy, Kcal/Kg	3,042	3,105	3,202
Crude protein, %	23.30	21.44	19.57
Ether extract, %	4.28	4.60	6.24
Crude fiber, %	2.64	2.55	2.63
Calcium, %	0.97	0.98	0.86
Available phosphorus, %	0.47	0.48	0.41
Lysine, %	1.38	1.22	1.10
Methionine, %	0.60	0.52	0.46

* Premix offered vitamins and minerals per Kg of diets: vitamin A, 9,800 IU; vitamin E, 50 IU; vitamin D3, 4,000 IU; vitamin K, 1.95 mg; vitamin B1, 1.3 mg; vitamin B12, 32 μg; vitamin B2, 27.3 mg; pantothenate, 13 mg; vitamin B3 nicotinic, 70 mg; vitamin B6, 4.6 mg; choline 1,200 mg; Zn (as zinc sulfate), 90 mg; biotin, 0.18 mg; Mn (as manganese sulfate), I (as potassium iodide), 80 mg; Cu (as copper sulfate), 10 mg; 65 mg; Fe (as ferrous sulfate), 0.50 mg; Se (as sodium selenite), 0.6 mg.

The feedstuff used in the experiment, as well as the experimental diets, underwent proximate chemical analysis to determine their moisture content, crude protein, and ether extract (30). This analysis was conducted following the guidelines of 30. Five experimental groups were conducted as follows: control (the birds were fed a basal diet and housed under thermoneutral conditions). The other four groups were housed under induced chronic heat stress conditions that comprised HS (the heat-stressed group was fed a basal diet without any additives), Resv-Lipo NPsI (heat-stressed birds were fed a basal diet supplemented with 50 mg/kg of resveratrol-loaded liposomal nanoparticles), Resv-Lipo NPsII (heat-stressed birds were fed a basal diet supplemented with 100 mg/kg of resveratrol-loaded liposomal nanoparticles), and Resv-Lipo NPsIII (heat-stressed birds were fed a basal diet supplemented with 150 mg/kg of resveratrol-loaded liposomal nanoparticles). The diet ingredients were thoroughly mixed and homogenized, and the Resv-Lipo NPs were spread uniformly over the feed at different levels.

### 2.3. Formulation and characterization of resveratrol-loaded liposomal nanoparticles

**Figure 1:**
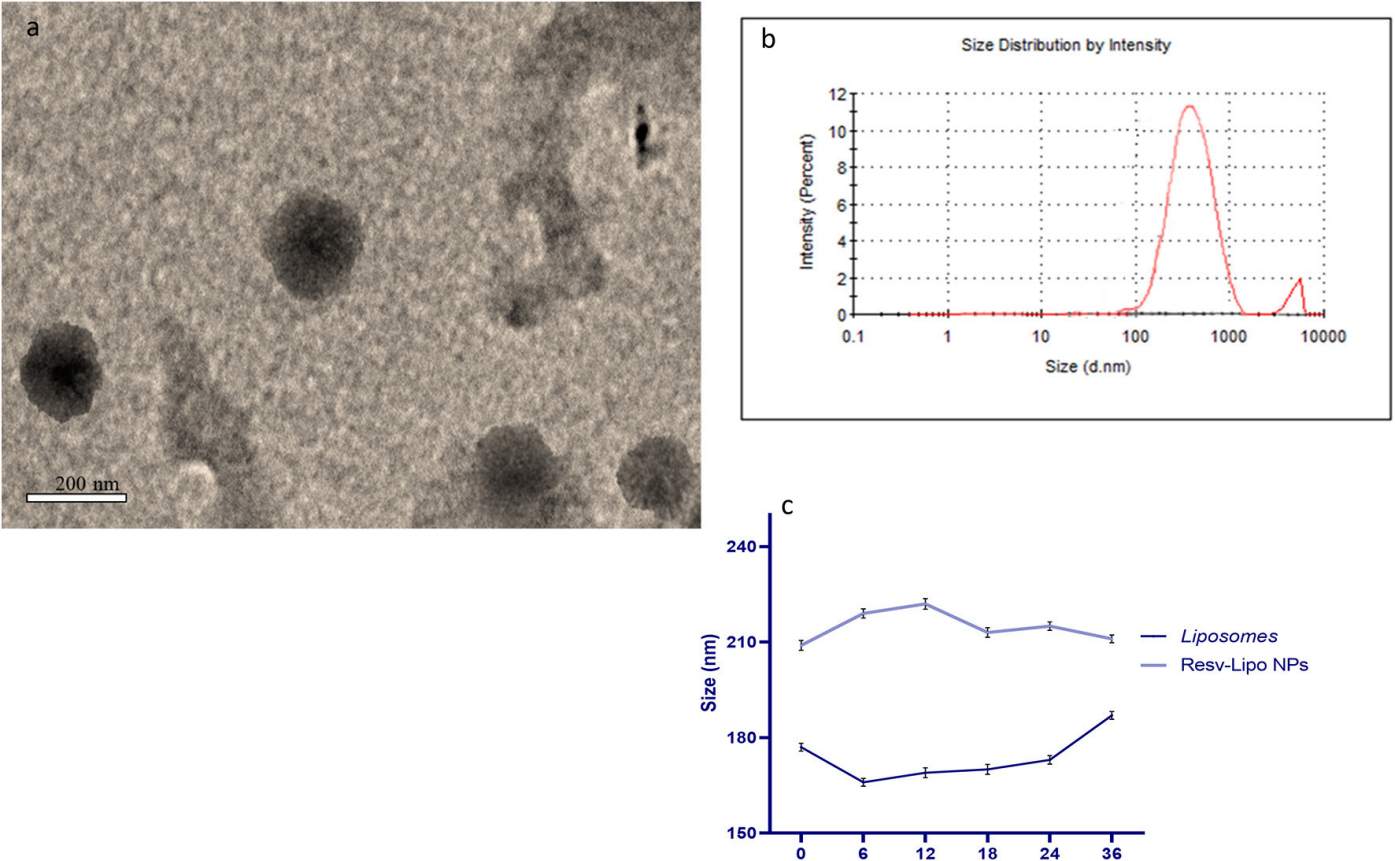
Transmission electron microscopy [TEM, **(a)**], Zeta potential and particles size of resveratrol loaded liposomes nano particles **(b)** and colloidal stability **(c)**.

Resveratrol (3,4,5-trihydroxy-trans-stilbene) and cholesterol (5a-cholestan-3ß-ol) were purchased from Sigma-Aldrich (No. PHR2201 and D6128, respectively). To prepare liposomes and resveratrol-loaded liposomes, the method described by Bonechi, Martini, Ciani, Lamponi, Rebmann, Rossi, and Ristori (19) was followed. The procedure involved mixing specific quantities of stock solutions in chloroform for lipids and ethanol for resveratrol, as outlined by Mohan et al. (31). A dry lipid film, either with or without resveratrol, was attained by vaporizing the solvents under a vacuum overnight and then hydrating it in phosphate-buffered saline at pH 7.4 and 60°C for 45 m. The resultant liposomal suspension was extruded carefully to obtain uniform liposomes following the procedure (19) and *via* a polycarbonate membrane with a 200-nm pore size (Liposofast Basic, Avestin, Canada). The prepared sample was diluted with deionized water, and then, a drop of the diluted sample was positioned on a carbon-copper grid and left to evaporate at room temperature. Morphological characterization of the prepared resveratrol-loaded nano-liposomes was determined using a transmission electron microscope (FE-TEM) (JEM 2100F, JEOL, Japan) working at an accelerating voltage of 200 kV (Figure 1a). Particle size and zeta potential analysis were performed using dynamic light scattering (Zeta Sizer, Malvern Instruments, UK) (Figure 1b). To measure the colloidal stability, the organized liposomes were kept undisturbed at 37°C. Samples (1 mL) were aliquoted and analyzed for their size after 0, 6, 12, 18, 24, and 36 h (Figure 1c).

### 2.4. Assessments of growth performance parameters

All individual birds reared under experimental conditions were weighed at 1,10, 24, and 42 days of age, and feed intake (g/bird) was estimated for each group to determine the body weight gain (BWG, g/bird), feed conversion ratio (FCR), relative growth rate (RGR), and protein efficiency ratio (PER) at the end of the starter, grower, and finisher stages, as described previously by Al-Khalaifah et al. (32), Farahat et al. (33), Ibrahim, Abdelfattah-Hassan, et al. (34), and Ibrahim, Ismail, et al. (35).

### 2.5. Sampling

On day 42, 10 birds per group were selected randomly to collect blood samples from wing veins. Blood samples were collected into a clean centrifuge tube without anticoagulant for the separation of serum following a centrifugation phase for 20 m at 3,000 rounds per min. The obtained sera were used for biochemical and antioxidant analyses. Then, the same birds were used for tissue sample collection after they were euthanized and eviscerated. Then, the liver, muscle, and jejunal samples were rapidly excised, flushed with ice-cold phosphate-buffered saline, and snap-frozen in liquid nitrogen for subsequent RNA isolation and gene expression. Additionally, breast muscle (~10 g, *n* = 10/group) was chilled at 4°C for 6 h and then immediately used for total antioxidant capacity (T-AOC) and malondialdehyde (MDA) evaluation.

### 2.6. Measurement of serum biochemical and hormone levels

Serum biochemical parameters, such as total protein, albumin, total cholesterol (TC), triacylglycerol (TAG), HDL-cholesterol, LDL-cholesterol, alanine aminotransferase (ALT), aspartate aminotransferase (AST), alkaline phosphatase (ALP), creatinine, and uric acid, were examined spectrophotometrically using a commercial diagnostic kit (Spinreact, S.A./S.A.U. Ctra. Santa Coloma, 7 E-17176 Sant Esteve De Bas, Spain). Serum triiodothyronine (T3), thyroxine (T4), and corticosterone levels were measured using the commercially available assay. Max Corticosterone ELISA kits were used for corticosterone level determination, and the procedures were performed according to the manufacturer's recommendation (Assay Pro-LLC, Saint Charles, Missouri, USA), while direct ELISA assay (Diametra Srl, Segrate, Italy) was used for T3 and T4 determination.

### 2.7. Antioxidant assessment

The serum concentration of malonaldehyde (MDA) level was assessed using the standard technique of NWLSS™ MDA assay kit NWK-MDA01 based on thiobarbituric acid. Based on the test kit directions, superoxide dismutase (SOD) was analyzed using a commercial assay kit (Sigma-Aldrich, 19160). Catalase (CAT) was determined spectrophotometrically using the NWLSS™ Catalase Activity Assay Kit Protocol NWK-CAT01. Glutathione peroxidase (GSH-Px) was measured using commercial assay kits from Sigma-Aldrich, G6137. Total antioxidant capacity (T-AOC) was assessed following the recommendation of the purchased kit (Nanjing Jiancheng Bioengineering Institute; Nanjing, China).

For the evaluation of oxidative stress-related markers in muscle, breast samples (*n* = 10 per group) were used. First, all observable connective and fat tissues were removed, and then, muscle tissues were homogenized with Tris- HCl buffer (1:10 w/v) and centrifuged for 10 min at 2500 x. Next, supernatants were used to assess T-AOC using the purchased kit (Nanjing Jiancheng Bioengineering Institute; Nanjing, China) and MDA using the NWLSS™ MDA assay kit NWK-MDA01.

### 2.8. Assessment of gene expression by quantitative real-time PCR

Total RNA was extracted from the breast muscles, the jejunal tissues, and the liver using the RNeasy Mini Kit; (Qiagen, Cat. No.74104), following the manufacturer's instructions, and then quantified using the NanoDrop^®^ ND-1000 Spectrophotometer (NanoDrop Technologies, Wilmington, NC, USA). The cDNA was synthesized using kits from RevertAidTM H Minus (Fermentas Life Science, Pittsburgh, PA, USA). One μL of this cDNA was mixed with 12.5 μL of 2× SYBR^®^ Green PCR mix with ROX from BioRad, 5.5 μL of RNase-free water, and 0.5 μL (10 pmol/μL) of each forward and reverse primer for each selected gene were added (Table 2). The real-time PCR amplification was accomplished using Rotor-Gene Q2 plex (Qiagen Inc., Valencia, CA, USA) with the following conditions: initial denaturation at 95°C for 10 min and 40 cycles at 95°C for 15 s and 60°C for 1 min. Relative fold changes in the expression of target genes were measured in triplicate and estimated using the comparative ^2−ΔΔ^Ct method with the GAPDH gene as an internal control to normalize target gene expression levels (36).

**Table 2:** Primers used for quantitative real-time PCR analysis.

**Gene**	**Primer sequence (5^′^-3^′^)**	**Accession number**
**Antioxidant related genes**		
*SOD*	F-GACGTGACAACACAGGTTGC R-TACAGCCACCGTAACAGCAG	XM_003449940.5
*CAT*	F-TCAGCACAGAAGACACAGACA R-GACCATTCCTCCACTCCAGAT	XM_031754288.1
*GSH-PX*	F-CCAAGAGAACTGCAAGAACGA R-CAGGACACGTCATTCCTACAC	NM_001279711.1
*HO-1*	F- ACTTCTATGGCAGCAACT R- AATAGCGGG GTAGGC	XM_205344.1
*Nr-f2*	F- ATCACGAGCCCTGAAACCAA R- GGCTGCAAAATGCTGGAAAA	NM_205117.1
**Heat shock proteins**		
*HSP-70*	F-TGGAGTCCTACGCCTTCAACA R-CAGGTAGCACCAGTGGGCAT	XM_003442456.5
*HSP-90*	F-GAGTTTGACTGACCCGAGCA R-TCCCTATGCCGGTATCCACA	NM_001109785.2
**Sirtuins**		
*SIRT-1*	F- AAGACCTGCTCCCAGAAACG R- ACAGCAAGGCGTGCATAGAT	NM_001004767.1
*SIRT-3*	F- ATCTTGTAGGACCGTTTGCC R- GCCAGCTGTCCTATTTGTCT	NM_001199493.1
*SIRT-7*	F- CCTGCGAAGTGGGTTACCTC R- GTCCCCTTCTCCCCAAAGTG	NM_001291971.1
**Muscle building**		
*mTOR*	F: CATGTCAGGCACTGTGTCTATTCTC R: CTTTCGCCCTTGTTTCTTCACT	XM_417614.5
*MSTN*	F: ATGCAGATCGCGGTTGATC R: GCGTTCTCTGTGGGCTGACT	NM_001001461.1
*MyoD*	F: CAGCAGCTACTACACGGAATCA R: GGAAATCCTCTCCACAATGCTT	NM_204214.2
**Cytokines**		
*IL-10*	F- CATGCTGCTGGGCCTGAA R-CGTCTCCTTGATCTGCTTGATG	NM_001004414.3
*IL-6*	F: AGG ACG AGA TGT GCA AGA AGTTC R: TTG GGC AGG TTG AGG TTG TT	NM_204628.1
*TNF-α*	F- CCCCTACCCTGTCCCACAA R- ACTGCGGAGGGTTCATTCC	XM_046900549.1
**Housekeeping**		
β-Actin	F-CAGCAAGCAGGAGTACGATG R-TGTGTGGTGTGTGGTTGTTTTG	XM_031749543.1
*GAPDH*	F: CAA CCC CCA ATG TCT CTG TT R: TCA GCA GCA GCC TTC ACT AC	NM205518

Superoxide dismutase: (SOD); catalase (CAT); glutathione peroxidase (GSH-PX); heme oxygenase-1 (HO-1); nuclear factor-erythroid 2-related factor-2 (Nr-f2); sirtuins (SIRT-1, SIRT-1and SIRT-1); heat shock protein-70 and 90 (HSP-70 And HSP-90); mechanistic target of rapamycin (mTOR); = myostatin (MSTN); = myogenic determination factor (MyoD); interleukin (IL-10: and IL−6); tumor necrosis factor-α (TNF-α); glyceraldehyde-3-phosphate dehydrogenase (GAPDH).

### 2.9. Histopathological examination

At the end of the experiment, the specimens from the liver tissues were collected from all experimental birds' necropsies, washed in phosphate-buffered saline, and fixed in freshly prepared neutral buffered formalin (10%). The fixed specimens were dehydrated in an ascending concentrated ethanol solution, cleared in xylene, implanted in paraffin wax, and cut into sections that were 5–7-μm thick and stained with hematoxylin and eosin (H & E), corresponding to the previous standard technique (37). The microphotographs were captured using a digital Dsc-W 130 super-steady Cyber-shot camera (Sony, Japan) linked to a light microscope (Olympus BX 21).

### 2.10. Statistical Assessment

All data were examined for normality and homogeneity using the tests of Kolmogorov–Smirnov and Levene, respectively. Statistical comparisons were made by the General Linear Model of Anova test *via* software SPSS version 21 for Windows (SPSS, Inc., Chicago, IL, USA) and the *post-hoc* Tukey test. The probability levels (*P* < 0.05) were determined to indicate statistical significance.

## 3. Results

### 3.1. Growth performance of birds under experimental conditions

The data regarding growth performance under normal and heat stress conditions are illustrated in Table 3. WE observed that birds exposed to heat-stressed conditions significantly displayed the lowest final weight gain and worst FCR (*P* < 0.05) (weight loss was ~20% vs. the thermoneutral group). In contrast, dietary supplementation with Resv-Lipo NPs at different levels reversed the negative outcomes (*P* > 0.05) in body gain, FCR, PER, and relative growth rate resulting from heat stress conditions in a dose-dependent manner. It is worth noting that heat-stressed birds that received Resv-Lipo NPs at the level of 150 mg/kg of diet exhibited significantly higher body weight gain and superior FCR (*P* < 0.05), which exceeded those in the thermoneutral group (increased by 5.8% vs. thermoneutral group). The total feed intake of birds in HS groups was significantly lowered (*P* < 0.05) except for the group supplemented with Resv-Lipo NPs at a level of 150 mg/kg when compared with the thermoneutral group.

**Table 3:** Effectiveness of Resv-Lipo NP supplementation at different levels on the growth performance of broiler chickens exposed to heat stress.

**Parameter**	**Control**	**HS**	**HS** + **Resv-Lipo NPs**			**SEM**	***p*-value**
			**I**	**II**	**III**		
Initial body weight, g	45.60	45.40	45.40	45.80	45.60	0.21	0.98
Final body weight, g/bird	2,597^b^	2,092^d^	2,381^c^	2,547^b^	2,747^a^	46.05	>0.001
Absolute body gain, g/bird	2,551^b^	2,047^d^	2,336^c^	2,501^b^	2,701^a^	46.03	>0.001
Total feed intake, g/bird	4,118^a^	3,691^d^	3,956^c^	3,992^bc^	4,095^ab^	33.73	>0.001
Feed conversion ratio	1.61^c^	1.80^a^	1.69^b^	1.60^c^	1.52^d^	0.02	>0.001
Protein efficiency ratio	3.28^b^	2.75^d^	3.13^c^	3.32^b^	3.49^a^	0.05	>0.001
Relative growth rate %	193.10^b^	191.50^d^	192.51^c^	192.93^b^	193.70^a^	0.14	>0.001

Control: the birds were fed a basal diet and housed under thermoneutral conditions, HS: the heat-stressed group fed a basal diet without any additives, HS+ resveratrol-loaded liposomal nanoparticles (Resv-Lipo NPs; I, II, and III): the heat-stressed birds were fed a basal diet supplemented with 50, 100, and 150 mg/kg of Resv-Lipo NPs, respectively. SEM: the standard error of the mean.

a,b,c,d Indicates that the same row carrying different superscripts is significantly different at a P-value of < 0.05.

### 3.2. Serum biochemicals and hormone levels

The results of biochemically related parameters and hormone levels (corticosterone, T3, and T4) are described in Table 4. TP and albumin serum concentrations were significantly reduced in the HS and HS+Resv-Lipo NPsI groups when compared with the thermoneutral group. Moreover, the HS group receiving Resv-Lipo NPsIII showed the highest TP, albumin, and globulin levels. Regarding the lipid profile, serum triglyceride, cholesterol, LDL, and VLDL levels were significantly (*p* < 0.05) reduced in the groups that received a higher dosage of Resv-Lipo NPs, regardless of HS. Furthermore, the levels of HDL reached their peak in HS birds that were fed 150 mg/kg of Resv-Lipo NPs. Notably, the levels of AST, ALT, ALP, and creatinine were significantly elevated in the HS birds, while the levels of AST, ALT, and ALP were restored in the control thermoneutral group, and the HS birds received different levels of Resv-Lipo NPs I, II and III. Additionally, the HS birds supplemented with Resv-Lipo NPsIII displayed a non-significant serum level (*p* > 0.05) of uric acid compared to the control thermoneutral group.

**Table 4:** Effectiveness of Resv-Lipo NP supplementation at different levels on the serum biochemicals and hormone levels of the broiler chickens exposed to heat stress.

**Parameter**	**Control**	**HS**	**HS+ Resv-Lipo NPs**			**SEM**	***p*-value**
			**I**	**II**	**III**		
**Serum proteins and lipid profile**							
Total protein, g/dl	3.49^b^	3.32^d^	3.38^c^	3.50^b^	3.77^a^	0.01	>0.001
Albumin, g/dl	1.88^b^	1.75^d^	1.80^c^	1.87^b^	1.93^a^	0.01	>0.001
Globulin, g/dl	1.61^b^	1.58^b^	1.58^b^	1.63^b^	1.83^a^	0.002	>0.001
Total cholesterol, mg/dl	124.45^b^	131.00^a^	122.12^c^	121.73^c^	119.74^d^	0.44	>0.001
Triacylglycerol, mg/dl	57.08^b^	60.67^a^	55.33^c^	54.87^cd^	54.54^d^	0.14	>0.001
HDL-cholesterol, mg/dl	96.03^c^	90.17^d^	98.80^b^	99.88^b^	102.92^a^	0.65	>0.001
LDL-cholesterol, mg/dl	17.00^b^	28.69^a^	12.26^c^	10.87^c^	5.92^d^	0.81	>0.001
VLDL-cholesterol, mg/dl	11.42^b^	12.14^a^	11.07^c^	10.97^cd^	10.91^d^	0.005	>0.001
**Liver and kidney function tests**							
ALT, U/L	17.92^b^	21.80^a^	18.22^b^	19.12^b^	18.06^b^	1.37	>0.001
AST, U/L	51.92^b^	56.06^a^	51.42^b^	52.44^b^	51.98^b^	1.43	>0.001
ALP, U/L	17.39^b^	24.36^a^	17.60^b^	17.81^b^	17.95^b^	0.58	>0.001
Creatinine, mg/ dl	0.80^b^	1.08^a^	0.86^b^	0.81^b^	0.83^b^	0.005	>0.001
Uric acid, mg/dl	2.61^d^	3.82^a^	3.42^ab^	3.18^bc^	2.77^cd^	0.08	>0.001
**Serum hormones**							
T3, nmol/L	3.92^a^	2.79^c^	3.48^b^	3.60^ab^	3.98^a^	0.06	>0.001
T4, nmol/L	18.86^a^	14.86^c^	17.27^b^	18.62^a^	19.01^a^	0.55	>0.001
Corticosterone, nmol/L	3.55^c^	7.13^a^	4.90^b^	3.92^c^	3.41^c^	0.140	>0.001

Control: the birds were fed a basal diet and housed under thermoneutral conditions, HS: the heat-stressed group fed a basal diet without any additives, HS + resveratrol-loaded liposomal nanoparticles (Resv-Lipo NPs; I, II, and III): the heat-stressed birds were fed a basal diet supplemented with 50, 100, and 150 mg/kg of Resv-Lipo NPs, respectively. HDL-cholesterol: high-density lipoprotein-cholesterol; LDL-cholesterol: low-density lipoprotein-cholesterol; VLDL-cholesterol: very low-density lipoprotein-cholesterol; ALT: Alanine aminotransferase; AST: aspartate aminotransferase; ALP: alkaline phosphatase; T3: triiodothyronine; T4: Thyroxin. SEM: the standard error of the mean.

a,b,c,d Indicates that the same row carrying different superscripts is significantly different at a P-value of < 0.05.

Notably, HS birds showed the lowest serum levels of corticosterone, T3, and T4 hormones. Meanwhile, serum levels of later hormones in the HS groups supplemented with dietary Resv-Lipo NPs at 100, and 150 mg/kg exhibited a non-significant value compared with the control thermoneutral group.

### 3.3. Measurements of serum and muscle antioxidant-related markers

Our data showed that the HS birds showed the lowest activities of serum antioxidant enzymes and levels of T-AOC, with significantly higher levels of MDA (Table 5). The highest activity of GSH-PX (*p* < 0.05) was detected in the serum of the HS group that received 150 mg/kg of Resv-Lipo NPs. Moreover, when subjected to heat stress, the group supplemented with Resv-Lipo NPs at the levels of 100 mg/kg, and 150 mg/kg showed a non-significant activity of serum catalase and SOD enzymes when compared with the thermoneutral control. The most significantly higher (*p* < 0.05) serum and muscular levels of T-AOC were distinctly identified in the HS group supplemented with 150 mg/kg of Resv-Lipo NPs. Supplementation with Resv-Lipo NPs, especially at higher levels (100 mg/kg and 150 mg/kg), significantly reduced serum and muscular levels of MDA.

**Table 5:** Effectiveness of Resv-Lipo NP supplementation at different levels on the serum and muscles antioxidant-related markers of the broiler chickens exposed to heat stress.

**Parameters**	**Control**	**HS**	**HS**+**Resv-Lipo NPs**			**SEM**	***p*-value**
			**I**	**II**	**III**		
**Serum antioxidants**							
SOD, U/mL	127.86^a^	121.93^c^	123.83^b^	127.70^a^	127.73^a^	0.47	>0.001
CAT, U/mL	14.04^a^	9.97^c^	11.37^b^	14.01^a^	14.30^a^	0.63	>0.001
GSH-PX, (nm(U/mL)	607.19^b^	536.00^d^	570.32^c^	606.92^b^	633.75^a^	67.80	>0.001
MDA, nmol/mL	1.56^c^	3.62^a^	2.49^b^	1.79^c^	1.27^d^	0.02	>0.001
T-AOC, ng/ml	0.72^b^	0.20^c^	0.31^c^	0.75^b^	0.92^a^	0.01	>0.001
**Antioxidants in breast muscles**							
MDA, nmol/mL	1.63^c^	3.70^a^	2.77^b^	1.86^c^	1.27^d^	0.18	>0.001
T-AOC, ng/ml	0.75^b^	0.22^d^	0.32^b^	0.73^c^	0.92^a^	0.06	>0.001

Control: the birds were fed a basal diet and housed under thermoneutral conditions, HS: the heat-stressed group a fed basal diet without any additives, HS+ resveratrol-loaded liposomal nanoparticles (Resv-Lipo NPs; I, II, and III): the heat-stressed birds were fed a basal diet supplemented with 50, 100, and 150 mg/kg of Resv-Lipo NPs, respectively. SOD: superoxide dismutase; CAT: catalase; GSH-PX: glutathione peroxidase; MDA: malondialdehyde; T-AOC: total antioxidant capacity. SEM: the standard error of the mean.

a,b,c,d Indicates that the same row carrying different superscripts is significantly different at a P-value of < 0.05.

### 3.4. mRNA expression levels of antioxidant-related genes

**Figure 2:**
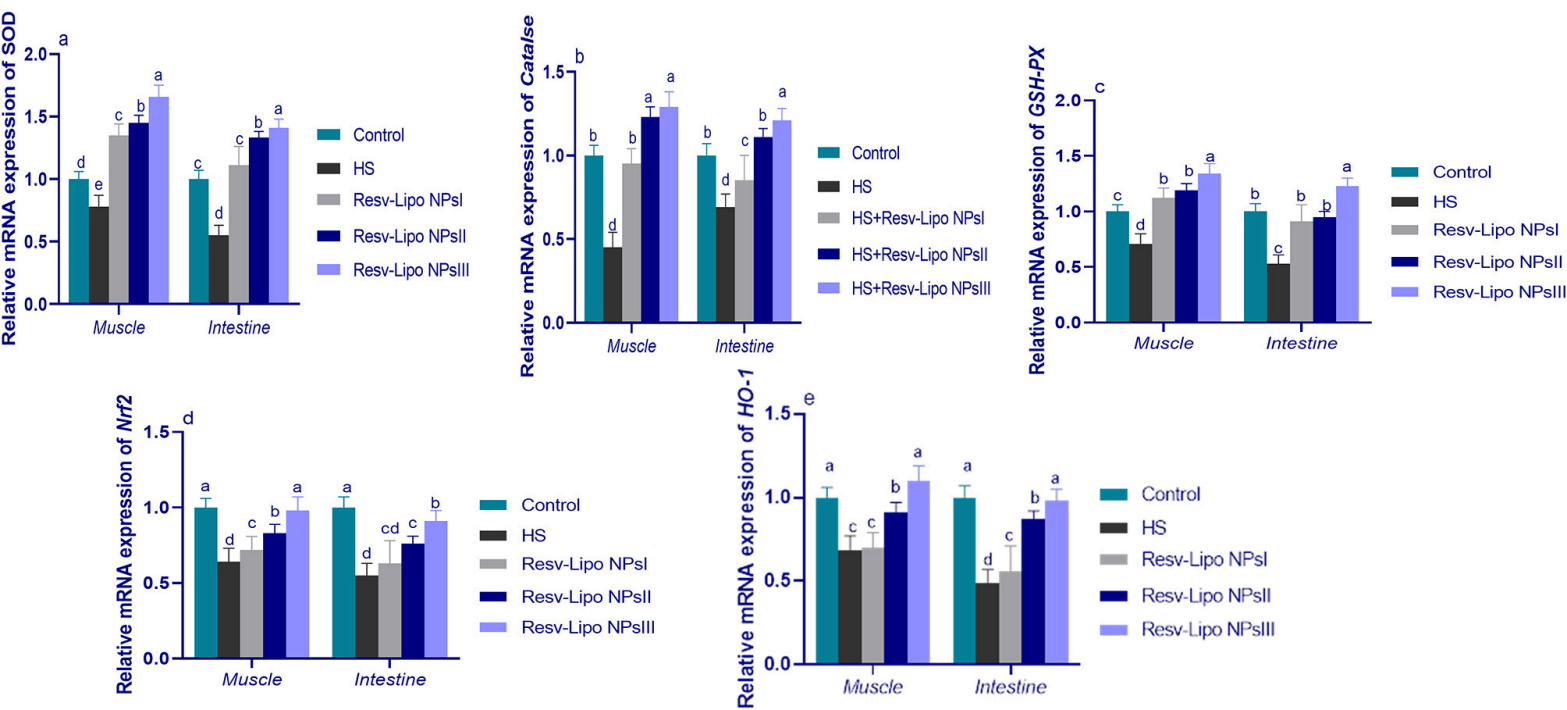
Effect of diets fortified with varying levels of Resv-Lipo NPs on the mRNA expression of genes related to antioxidants {superoxide dismutase [*SOD*, **(a)**], catalase [*CAT*, **(b)**], glutathione peroxidase [*GSH*-*PX*, **(c)**], nuclear factor-erythroid factor 2-related factor 2 [*Nrf2*, **(d)**], and heme oxygenase-1 [*HO-1*, **(e)**] in the breast muscle and the jejunal tissues of the broiler chickens exposed to heat stress. Control: the birds fed a basal diet and housed under thermoneutral conditions, HS: the heat-stressed group fed a basal diet without any additives, HS+ resveratrol-loaded liposomal nanoparticles (Resv-Lipo NPs; I, II, and III): the heat-stressed birds were fed a basal diet supplemented with 50, 100, and 150 mg/kg of Resv-Lipo NPs, respectively. ^abcd^Indicates that the same columns carrying various superscripts are significantly different at *P* < 0.05. Data are presented as means ± SE.

Exposure to HS dramatically decreased the intestinal and muscular expression of antioxidant-regulated genes (Figure 2). Intestinal and muscular upregulation of *CAT* and *SOD* expression was more prominent (*p* < 0.05) in the group supplemented with higher levels of Resv-Lipo NPs. Notably, the HS group supplemented with Resv-Lipo NPsIII showed the most prominent upregulation of the *GSH-PX* gene in both the intestine and the muscle regardless of heat stress (increases of up to 1.23 and 1.34-fold, respectively, when compared to the thermoneutral group). Moreover, the downregulation of *Nrf2* and *HO-1* in the HS group was inversely upregulated after the dietary inclusion of Resv-Lipo NPs, especially at higher levels.

### 3.5. mRNA expression levels of heat shock proteins and sirtuin's regulatory genes

**Figure 3:**
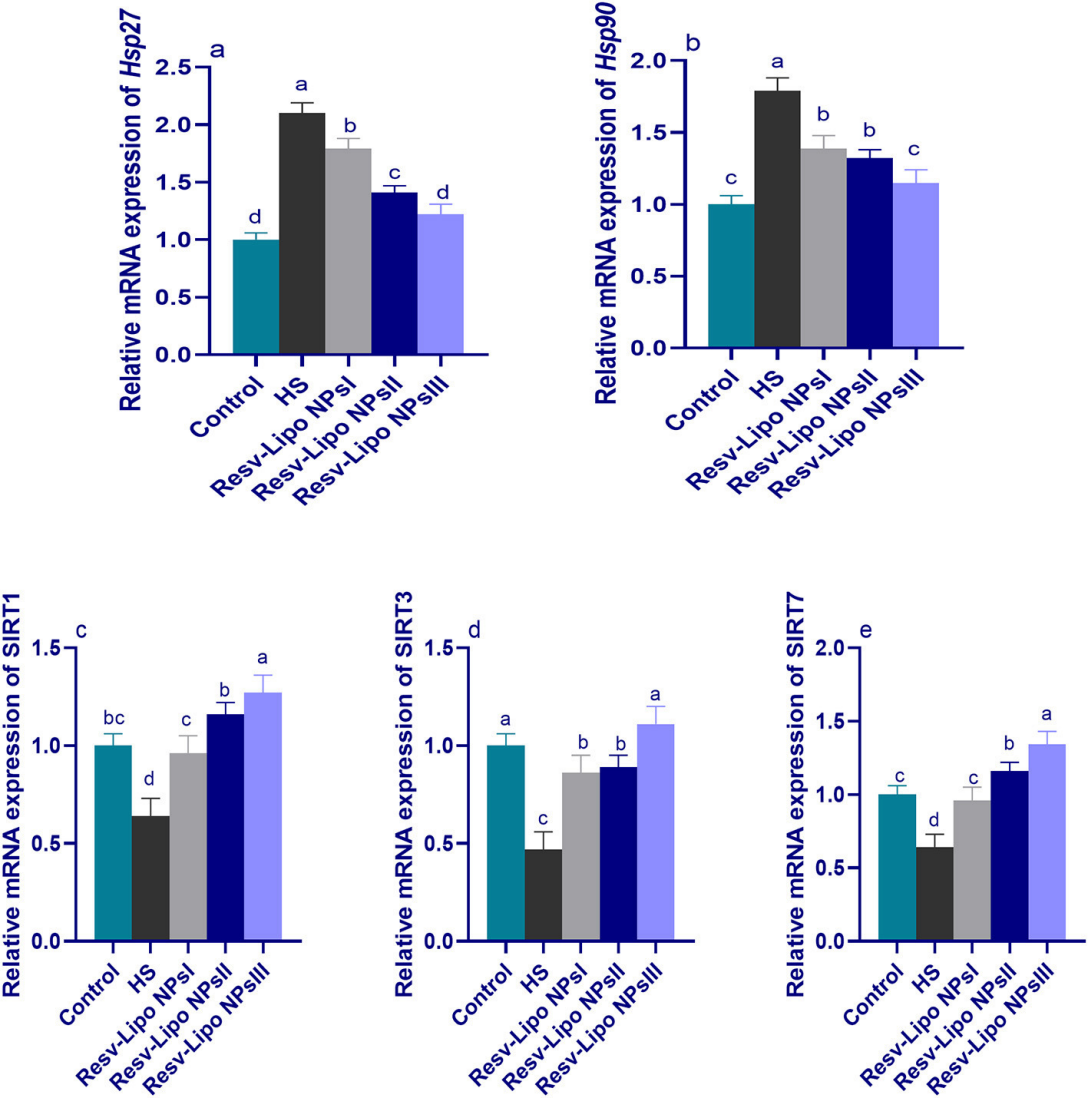
Effect of diets fortified with varying levels of Resv-Lipo NPs on the mRNA expression of HSP [*Hsp27* **(a)** and *Hsp90*, **(b)**] and the downregulation of sirtuins [*SIRT1* **(c)**, *SIRT3* **(d)**, and *SIRT7* **(e)**] in the broiler chickens exposed to heat stress. Control: the birds fed a basal diet and housed under thermoneutral conditions, HS: the heat-stressed group fed a basal diet without any additives, HS+ resveratrol-loaded liposomal nanoparticles (Resv-Lipo NPs; I, II, and III): the heat-stressed birds fed a basal diet supplemented with 50, 100, and 150 mg/kg of Resv-Lipo NPs, respectively. ^abcd^Indicates that the same columns carrying various superscripts are significantly different at *P* < 0.05. Data are presented as means ± SE.

Figure 3 shows greater upregulation of HSP (Hsp27 and Hsp90) and downregulation of Sirtuins (*SIRT1, SIRT3*, and *SIRT7*) in the HS group. It was noticed that the expression of SIRT3 was significantly upregulated in the HS group supplemented with Resv-Lipo NPs. Furthermore, no significant differences were detected between the control thermoneutral group and the HS-supplemented Resv-Lipo NPs group at a dose of 150 mg/kg.

Regarding *SIRT1* and *SIRT7*, the maximum expression levels were observed to be highest at 100 mg/kg and 150 mg/kg, respectively, showing an increase of 1.27 and 1.34-fold, respectively, compared to the control thermoneutral group. Notably, the higher expression levels of *Hsp2*7 and *Hsp90* in the HS-induced group were downregulated (*p* < 0.05) in those supplemented with Resv-Lipo NPs in a dose-dependent manner.

### 3.6. mRNA expression levels of myogenic regulatory factors

**Figure 4:**
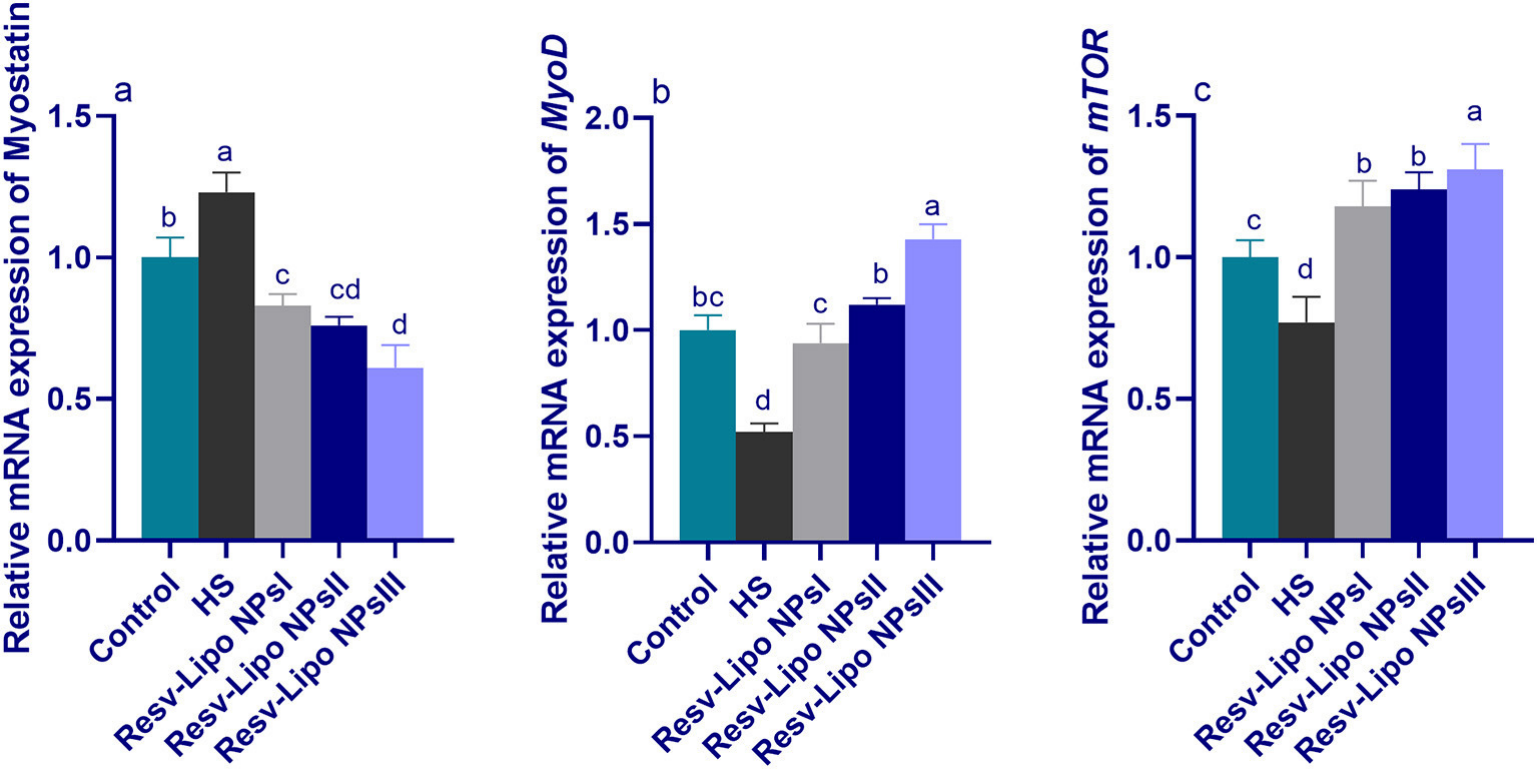
Effect of diets fortified with varying levels of Resv-Lipo NPs on the mRNA expression of myogenic regulatory factors {myostatin **(a)**, myoblast determination protein 1 [*MyoD*, **(b)**] and mammalian target of rapamycin [*mTOR*, **(c)**]} in the breast muscles of broiler chickens exposed to heat stress. Control: the birds fed a basal diet and housed under thermoneutral conditions, HS: the heat-stressed group fed a basal diet without any additives, HS+ resveratrol-loaded liposomal nanoparticles (Resv-Lipo NPs; I, II, and III): the heat-stressed birds fed a basal diet supplemented with 50, 100, and 150 mg/kg of Resv-Lipo NPs, respectively. ^abcd^Indicates that the same columns carrying various superscripts are significantly different at (*P* < 0.05). Data are presented as means ± SE.

As displayed in Figure 4, compared to the thermoneutral group, the mRNA expressions of myostatin in the breast muscle were upregulated. Inversely, the mRNA expression levels of *MyoD* and *mTOR* (*p* < 0.05) were downregulated in the HS group. In contrast, the birds exposed to heat stress and fed Resv-Lipo NPs showed a downregulated expression (*p* > 0.05) of myostatin with increasing levels. Moreover, the highest expression levels of *mTOR* were detected in the group fed with 150 mg/kg Resv-Lipo NPs (up to 1.30-fold compared to the control group). In addition, in the HS-induced group, Resv-Lipo NPs supplemented with one at 150 mg/kg exhibited the maximum expression level of MyoD.

### 3.7. mRNA expression levels of immune regulatory genes

**Figure 5:**
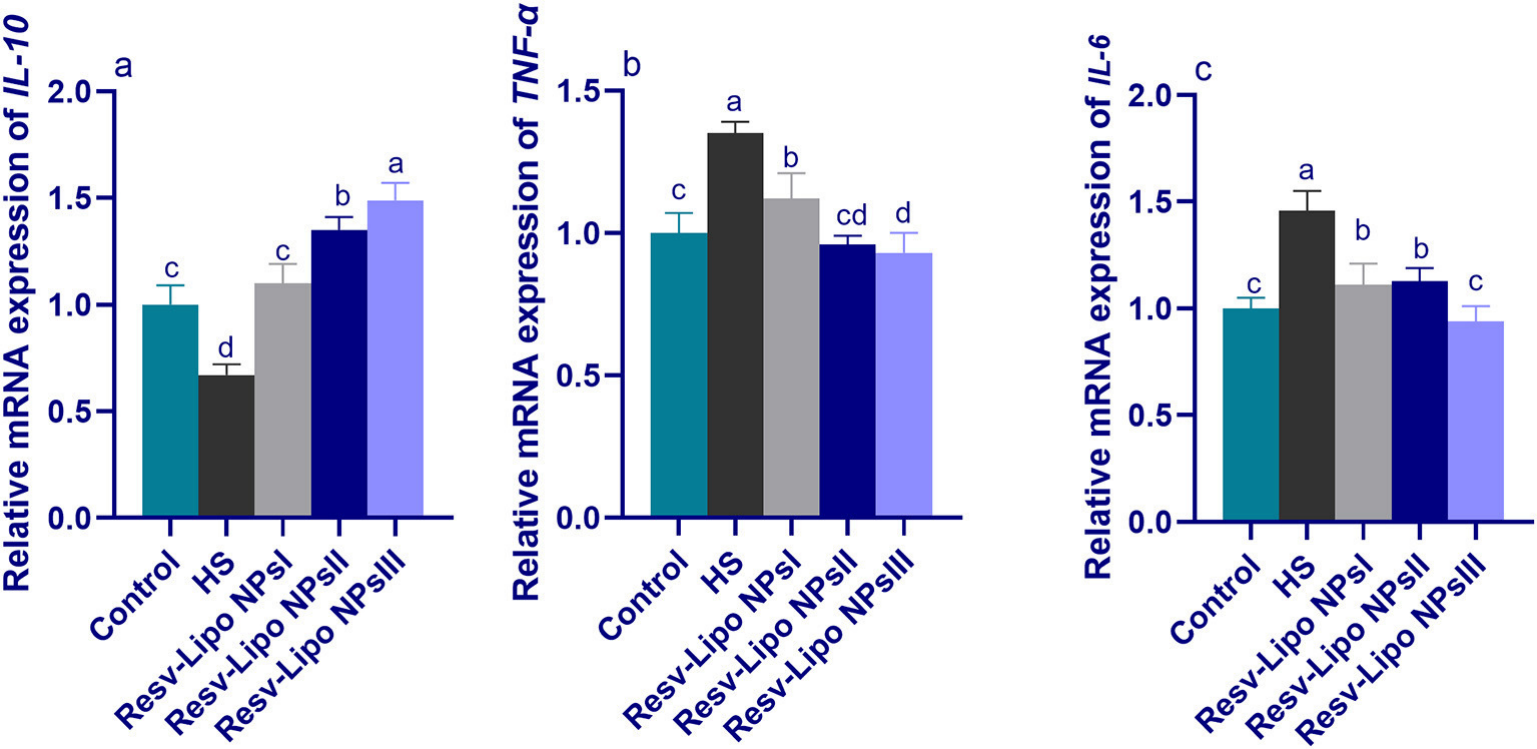
Effect of diets fortified with varying levels of Resv-Lipo NPs on the mRNA expression of immune-linked genes {interleukin [*IL-10*, **(a)**], tumor necrosis factor [*TNF-*α, **(b)**], and *IL-6*, **(c)**} in the breast muscles of broiler chickens exposed to heat stress. Control: the birds fed a basal diet and housed under thermoneutral conditions, HS: the heat-stressed group fed a basal diet without any additives, HS+ resveratrol-loaded liposomal nanoparticles (Resv-Lipo NPs; I, II, and III): the heat-stressed birds fed a basal diet supplemented with 50, 100, and 150 mg/kg of Resv-Lipo NPs, respectively. ^abcd^Indicates that the same columns carry various superscripts that are significantly different at *P* < 0.05. Data are presented as means ± SE.

As shown in Figure 5, increasing the inclusion levels of dietary Resv-Lipo NPs significantly upregulated the expression levels of *IL-10* in a dose-dependent manner. Excessive immune response, reflected by higher expression of *TNF-*α and *IL-6* in the HS group, was declined in the Resv-Lipo NP-supplemented groups. Moreover, there were no significant differences in the expression of *TNF-*α and *IL-6 between the* HS group supplemented with 150 mg/kg of Resv-Lipo NPs and the control thermoneutral group.

### 3.8. Histopathological outcomes

**Figure 6:**
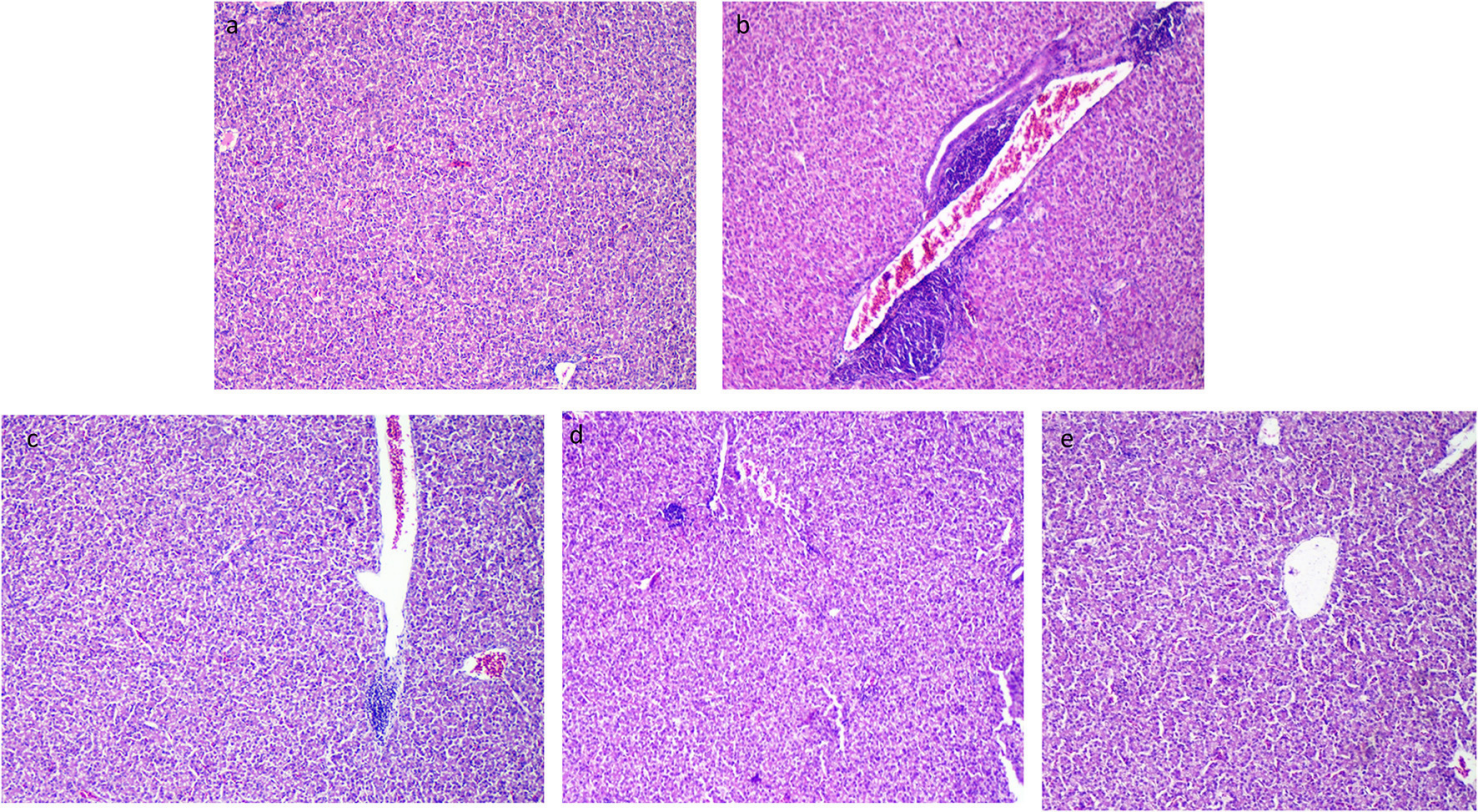
Effect of diets fortified with varying levels of Resv-Lipo NPs on histopathological alterations of the liver tissues post-heat stress exposure. **(a)** Negative control: the birds fed a basal diet without exposure to heat stress; **(b)** positive control: the birds fed a basal diet and exposed to heat stress; **(c)** the birds fed a basal diet exposed to heat stress and supplemented with Resv-Lipo NPsI, **(d)** the birds fed a basal diet exposed to heat stress and supplemented with Resv-Lipo NPsII; and **(e)** the birds fed a basal diet exposed to heat stress and supplemented with Resv-Lipo NPsIII. The liver tissues of the control group (thermoneutral group) presented normal hepatic parenchyma, acini, and a blood vessel tree **(a)**. Perivascular minute aggregations of inflammatory cells were visible in the Resv-Lipo NPs I and, to a lesser extent, in the Resv-Lipo NPs II supplemented group **(c, d)**. In the Resv-Lipo NPs III group, the liver revealed an apparent normal hepatic parenchyma, blood vessels, and hepatocytes **(e)**.

The liver tissues of the control group (the thermoneutral group) presented normal hepatic parenchyma, acini, and a blood vessel tree (Figure 6a). After exposure to heat stress, the histopathological architecture of unsupplemented Resv-Lipo NPs (the HS control) showed broad lesions comprising congested blood vessels at the portal area, hydropic degeneration in hepatocytes, and focal areas of leukocytic infiltration (Figure 6b). Resv-Lipo NP supplementation nearly restored the normal liver histological architecture (Figures 6c–e), and this was achieved by increasing the Resv-Lipo NPs dosage. Perivascular minute aggregations of inflammatory cells were still visible in the Resv-Lipo NPs I and to a lesser extent in the Resv-Lipo NPs II supplemented group, but to a lesser extent compared to challenged non-supplemented chicks (Figures 6c, d). Meanwhile, in the Resv-Lipo NPs III group, the liver revealed normal hepatic parenchyma, blood vessels, and hepatocytes (Figure 6e).

## 4. Discussion

Stress conditions associated with elevated environmental temperatures and high stocking densities can negatively affect the survival and performance of broiler chickens. These conditions are also detrimental to the economics of modern poultry farms. Heat stress, in particular, can adversely affect weight gain and deteriorate broiler chickens' feed efficiency due to oxidative imbalance, metabolic disorders, and immune suppression (2, 38, 39). Oxidative damage is the main negative effect of heat stress. With the excessive accumulation of ROS, damage to the intestinal mucosa and muscle protein hydrolysis can occur (40), worsening the birds' performance (41). Therefore, using exogenous natural antioxidants can help birds restore oxidative balance and maintain superior growth performance during periods of heat stress. In the search for polyphenolic compounds with strong antioxidant capacity, trans-resveratrol has been identified as a promising candidate; however, its low bioavailability has limited its use in the biological system. The current study demonstrated that nanoliposomal carriers formulated with trans-resveratrol could be targeted for their efficient use as new dietary feed additives that counteract the heat stress conditions in poultry farms. The birds exposed to the induced heat stress and those who received different levels of resveratrol-loaded liposomal nanocarriers (Resv-Lipo NPs) restored an efficient level of performance compared to the heat stress-induced group. In previous studies, adding resveratrol partially restored the unfavorable effects of heat stress on broiler chickens' growth rate (39, 42).

Moreover, Liu et al. (18) stated that resveratrol could play a crucial role as a feed additive to monitor the negative consequences of heat stress on the growth performance of broiler chickens (18). Notably, the birds exposed to heat stress and those who received higher supplementation levels of Resv-Lipo NPs maintained the highest growth rate and feed efficiency compared to the control thermoneutral group. The significant superior growth performance of broiler chickens following dietary administration of Resv-Lipo NPs, unlike free Resv in previous studies, can be related to Resv incorporation into an excellent nano-candidate that enhanced its functions of scavenging harmful free oxygen and lipid radicals resulting from induced heat stress conditions (18, 19).

Exposure to chronic and uncontrollable heat stress may disrupt the balance between antioxidant defense mechanisms and oxidative stress by depleting enzymatic antioxidants and elevating lipid peroxidation (43). It has been recognized that superfluous ROS triggered by heat stress can cause oxidative injury, such as lipid peroxidation and oxidative damage to proteins and DNA (44). Additionally, heat stress has been shown to reduce nutrient digestibility, probably due to too much ROS production that oxidizes and damages the intestinal tissues (45). Excessive temperatures can lead to alterations in cell oxidative status and ROS accumulation with low production levels of T-AOC, T-SOD, and GSH-Px (46) and the overproduction of MDA (47). Excessive ROS can cause cholesterol peroxidation, reducing membrane fluidity and receptor activity, thereby impairing membrane function (48). Considering the antioxidant status of the birds after heat stress exposure, a dose-dependent increase in T-AOC and serum antioxidant enzyme activities and a good upregulation of the expression of antioxidant enzyme-related genes in muscle and intestine were noted in all bird groups, which received different supplementation levels of Resv-Lipo NPs.

Moreover, the birds that received Resv-Lipo NPs had significantly reduced levels of MDA, which reflected the reduction of lipid peroxidation under heat stress-induced reactive oxygen species in the broiler chickens. In the same context, resveratrol displayed antioxidant properties that prevented glutathione disulfide formation and sustained glutathione in a reduced form, inhibiting the cellular damage generated by free radical reactions (11). Furthermore, it augmented the expression of antioxidant-related genes and assisted in the inactivation and elimination of oxidative precursors (12, 18, 49), suggesting its protective role against oxidative stress caused by heat stress. Resveratrol works to eliminate free radicals and ROS due to its possession of a phenyl group capable of giving an electron to free radicals and ROS, thus reducing oxidative stress (50). Nrf2 acts as a transcription factor to control antioxidant-related gene expression, which is vital for sustaining redox balance, such as SOD, GPX, HO-1, and CAT (51, 52). Our study showed that heat stress downregulated the expression of Nrf2 and HO-1, whereas the contra administration of higher levels of Resv-Lipo NPs restored the expression levels of Nrf2 and HO-1 to nearly the same level as the control thermoneutral group. In another study, it was found that dietary supplementation with 400 mg/kg of free Resv supplementation reduced oxidative stress in fatty liver hemorrhagic syndrome by boosting the activity of antioxidant enzymes (Nrf2, SOD-1, and HO-1) (53). All these findings suggested the prospective role of dietary Resv-Lipo NPs in activating the antioxidant defense mechanism and promoting overall body health. Similarly, the incorporation of RESV into lipid nanosystems as liposomes has an inhibitory impact on lipid peroxidation, COX activity, and NO production, which in turn indicates its anti-inflammatory and anti-oxidant effects (20). Additionally, the radical scavenging effect of Resv was enhanced with liposomal systems, as evinced by reducing the ROS production during spontaneous oxidative stress (54). The scavenging effect of RESV on ROS was enhanced *via* its incorporation into liposomal nanocarriers, which resulted in ameliorating tissue damage in response to oxidative stress (55).

Increased serum corticosterone concentration is considered a marker of heat stress (4). The birds subjected to heat stress showed higher serum corticosterone concentrations compared to the neutral thermoregulated group. In contrast, the administration of Resv-Lipo NPs in a dose-dependent manner greatly reduced their concentrations in serum, suggesting their ability to reduce the overexcitement of the hypothalamic-pituitary-adrenal axis in response to heat stress. Additionally, thyroid hormones are vital for maintaining animal homeostasis (56). The reduction in the T3 and T4 levels in the heat-stressed birds supplemented with Resv-Lipo NPs, especially at higher doses, indicated their promosing role regulating the hypothalamic-pituitary-thyroid axis and mitigating stressors, which is in line with the results of Meng, Deng, Xiao, Arowolo, Liu, Chen, Deng, He, and He (56). Numerous investigations have shown an inverse association between dietary bioactive substances with antioxidant potential and HSP expression (57, 58). Heat shock proteins, comprising Hsp27, Hsp70, and Hsp90, play a critical protective role in sustaining the metabolic and structural integrity of the organ against stress-induced tissue damage (59). In this study, the expression of HSP was downregulated after exposure to chronic heat stress, and feeding on Resv-Lipo NPs, even at lower levels, indicated their role in attenuating the negative impacts of heat stress. Similarly, resveratrol has been demonstrated to influence the expression of HSP27, HSP70, and HSP90 in the thymus and the spleen of chickens (17, 18). Sirtuin (SIRTs) enzymes and the expression of their related genes play an important role in glucogenesis and fat oxidation, as well as the modulation of oxidative stress-related processes and functions, including DNA repair and metabolic functions (60). The sirtuin family, which includes SIRTs 1–7, SIRT1, SIRT3, and SIRT5, is involved in redox regulation and helps to protect the cell from ROS. Moreover, SIRT2, SIRT6, and SIRT7 modulate key oxidative stress genes (60). SIRT1 was shown to play a crucial role in preventing oxidative damage, which is essential for DNA repair after H_2_O_2_-induced damage (60, 61). Moreover, treatment with the SIRT1 activator resveratrol inhibited H2O2-induced cell death and reduced cell proliferation. On the other hand, the SIRT1 inhibitors were shown to augment H_2_O_2_-induced cell death (62, 63). Moreover, SIRT3 and SIRT7 have also been revealed to intermediate enzyme deacetylation, which is responsible for the reduction of ROS, leading to protection against oxidative stress-dependent developments and disorders such as cardiac hypertrophy and dysfunction, cancer, aging, and neural degeneration (64, 65). Another compelling result of the present study is that the expression levels of SIRT1, SIRT3, and SIRT7 were upregulated even in the presence of oxidative stress. These novel findings suggested that other oxidative stress-linked activities of Resv-Lipo NPs may be due to their role in stimulating sirtuin-related gene expression. The essence of muscle growth is protein accumulation, and the balance between the rates of protein synthesis and protein catabolism affects muscle mass (66, 67). It has been reported that HS reduces skeletal muscle protein deposition in broiler chickens (68). However, the exact mechanism that controlled this reduction remains poorly understood. It has recently been found that constant heat stress can lead to skeletal muscle protein breakdown *via* increased mRNA expression levels of myostatin and decreased expression of *MyoD* and *mTOR* (69). Inappropriate ROS concentrations during heat stress periods can damage skeletal muscle and inhibit protein synthesis (70). Excessive ROS in myoblasts can promote nuclear factor kappa B (NF-κB) and reduce MyoD expression level, thereby preventing myogenic differentiation (71). This study's findings suggest that, when subjected to heat stress, birds that received higher levels of Resv-Lipo NPs exhibited a downregulated level of the myostatin gene, which, in turn, upregulated the expression level of the *MyoD* gene in the breast muscle. This could be attributed to the potential indirect role of Resv-Lipo NPs in restoring muscle myogenesis by reducing the elevated ROS levels that induced inflammatory changes and inhibited myogenesis in the muscle, affecting its growth. A dietary supplement with antioxidant capacity can potentiate muscle growth by balancing its redox hemostasis (72, 73). Moreover, in this study, the incorporation of resveratrol in a liposomal nanocarrier controlled its release and increased its bioavailability, thus intensifying its mode of action (19). Oxidative stress can provoke inflammation, as reflected in the excessive expression of pro-inflammatory cytokines, such as *IL-6* and *TNF-*α (74, 75). It has recently been reported that Resv (400 mg/kg) supplementation decreased oxidative stress in fatty liver hemorrhagic syndrome by reducing inflammatory cytokines (*IL-6, NF-*κ*B*, and *TNF-*α) and mRNA expression in the ovaries (53). In this regard, Yang et al. (76) also described that feeding ducks dietary Resv at the level of 400 mg/kg protected against inflammation-induced injury resulting from heat stress, as indicated by decreasing IL-6 secretion in the jejunum. In this study, the results related to the expression of proinflammatory cytokines after exposure to HS indicated that feeding lower doses of Resv-Lipo NPs compared to free Resv in other studies was more effective in minimizing excessive inflammatory reactions, resulting from oxidative stress.

High environmental temperatures stimulated oxidative damage in the liver tissues of broiler chickens, which further resulted in lipid metabolism abnormalities (77). In the current study, exposure to chronic heat stress led to significant alternations in the normal architecture of the liver, resulting in prominent hepatic damage, which is consistent with the findings of Tang et al. (78). However, post-supplementation with Resv-Lipo NPs resulted in a dose-dependent improvement in the histopathological architecture of the liver. The best results in this study were obtained from the Resv-Lipo NPs III group, followed by the percentages of the Resv-Lipo NPsII and Resv-Lipo NPsI groups. Feeding broiler chickens exposed to chronic heat stress with free resveratrol (at a level of 400 mg/kg) efficiently reduced liver injury and alleviated liver cell apoptosis and damage (79). Notably, our results showed that groups exposed to HS and treated with Resv-Lipo NPs showed better recovery from liver damage, even at lower doses, compared to previous studies that used higher levels of free resveratrol (18, 42). This improved efficacy could be attributed to the effective delivery of Resv through its incorporation into a liposomal nanocarrier, which ensures better functionality of the compound.

## 5. Conclusion

The use of liposomes as an efficient nanocarrier for resveratrol delivery was shown to enhance its bioactivity and stability. The boosted mode of action of Resv-Lipo NPs enables their protection from environmental and gut conditions until they reach the target tissues. These findings are supported by the superior growth performance of broiler chickens fed Resv-Lipo NPs despite exposure to induced HS, as Resv-Lipo NPs alleviate oxidative damage. Molecular-based studies further strengthen the potential role of Resv-Lipo NPs in modulating the expression of myogenic regulatory factors responsible for muscle growth, HSP, and the sirtuin family. Overall, the current study proved, for the first time, that Resv-Lipo NPs efficiently mitigate heat stress-associated disorders at lower concentrations.

## Data availability statement

The original contributions presented in the study are included in the article/supplementary material, further inquiries can be directed to the corresponding author.

## Ethics statement

The animal study was reviewed and approved by Animal Care and Use Committee (ZU-IACUC/2/f/201/2022), Poultry Research Unit, Faculty of Veterinary Medicine, Zagazig University.

## Author contributions

All authors contributed to the study's design, methodology, data collection and analysis, statistical analysis, and manuscript writing. All authors contributed to the article and approved the submitted version.

## References

[1] Smith P Gregory PJ. Climate change and sustainable food production. Proc Nutr Soc. (2013) 72:21–8. 10.1017/S0029665112002832 23146244

[2] Ghazi S Habibian M Moeini M Abdolmohammadi A. Effects of different levels of organic and inorganic chromium on growth performance and immunocompetence of broilers under heat stress. Biol Trace Elem Res. (2012) 146:309–17. 10.1007/s12011-011-9260-1 22127829

[3] Mashaly M Hendricks G 3rd Kalama M Gehad A Abbas A Patterson P. Effect of heat stress on production parameters and immune responses of commercial laying hens. Poult Sci. (2004) 83:889–94. 10.1093/ps/83.6.889 15206614

[4] Quinteiro-Filho W Ribeiro A Ferraz-de-Paula V Pinheiro M Sakai M Sá L Ferreira A Palermo-Neto J. Heat stress impairs performance parameters, induces intestinal injury, and decreases macrophage activity in broiler chickens. Poult Sci. (2010) 89:1905–14. 10.3382/ps.2010-00812 20709975

[5] Gu X Hao Y Wang X. Overexpression of heat shock protein 70 and its relationship to intestine under acute heat stress in broilers: 2. Intestinal oxidative stress. Poult Sci. (2012) 91:790–9. 10.3382/ps.2011-01628 22399716

[6] Hu R He Y Arowolo MA Wu S He J. Polyphenols as potential attenuators of heat stress in poultry production. Antioxidants. (2019) 8:67. 10.3390/antiox8030067 30889815 PMC6466569

[7] Ibrahim D Moustafa A Metwally AS Nassan MA Abdallah K Eldemery F. Potential application of cornelian cherry extract on broiler chickens: growth, expression of antioxidant biomarker and glucose transport genes, and oxidative stability of frozen meat. Animals. (2021) 11:1038. 10.3390/ani11041038 33917066 PMC8067757

[8] Ibrahim D Sewid AH Arisha AH Abd El-Fattah AH Abdelaziz AM Al-Jabr OA. Influence of glycyrrhiza glabra extract on growth, gene expression of gut integrity, and campylobacter jejuni colonization in broiler chickens. Front Vet Sci. (2020) 7: 612063. 10.3389/fvets.2020.612063 33415133 PMC7782238

[9] Zhang C Wang L Zhao X Chen X Yang L Geng Z. Dietary resveratrol supplementation prevents transport-stress-impaired meat quality of broilers through maintaining muscle energy metabolism and antioxidant status. Poult Sci. (2017) 96:2219–25. 10.3382/ps/pex004 28339929 PMC5850463

[10] Zhang C Luo J Yu B Chen J Chen D. Effects of resveratrol on lipid metabolism in muscle and adipose tissues: a reevaluation in a pig model. J Funct Foods. (2015) 14:590–5. 10.1016/j.jff.2015.02.039

[11] Hung L-M Chen J-K Huang S-S Lee R-S Su M-J. Cardioprotective effect of resveratrol, a natural antioxidant derived from grapes. Cardiovasc Res. (2000) 47:549–55. 10.1016/S0008-6363(00)00102-4 10963727

[12] Yan Y Yang J-Y Mou Y-H Wang L-H Zhou Y-N Wu C-F. Differences in the activities of resveratrol and ascorbic acid in protection of ethanol-induced oxidative DNA damage in human peripheral lymphocytes. Food Chem Toxicol. (2012) 50:168–74. 10.1016/j.fct.2011.10.046 22056336

[13] Houtkooper RH Pirinen E Auwerx J. Sirtuins as regulators of metabolism and healthspan. Nat Rev Mol Cell Biol. (2012) 13:225–38. 10.1038/nrm3293 22395773 PMC4872805

[14] Satoh A Stein L Imai S. The role of mammalian sirtuins in the regulation of metabolism, aging, and longevity. Histone Deacetylases Biol Clin Impl. (2011) 206:125–62. 10.1007/978-3-642-21631-2_7 21879449 PMC3745303

[15] Guarente L. Sirtuins, aging, and medicine. N Engl J Med. (2011) 364:2235–44. 10.1056/NEJMra1100831 21651395

[16] Jeśko H Wencel P Strosznajder RP Strosznajder JB. Sirtuins and their roles in brain aging and neurodegenerative disorders. Neurochem Res. (2017) 42:876–90. 10.1007/s11064-016-2110-y 27882448 PMC5357501

[17] Liu L Fu C Yan M Xie H Li S Yu Q. Resveratrol modulates intestinal morphology and HSP70/90, NF-κB and EGF expression in the jejunal mucosa of black-boned chickens on exposure to circular heat stress. Food Funct. (2016) 7:1329–38. 10.1039/C5FO01338K 26843443

[18] Liu L He J Xie H Yang Y Li J Zou Y. Resveratrol induces antioxidant and heat shock protein mRNA expression in response to heat stress in black-boned chickens. Poult Sci. (2014) 93:54–62. 10.3382/ps.2013-03423 24570423

[19] Bonechi C Martini S Ciani L Lamponi S Rebmann H Rossi C Ristori S. Using liposomes as carriers for polyphenolic compounds: the case of trans-resveratrol. PLoS ONE. (2012) 7:e41438. 10.1371/journal.pone.0041438 22936976 PMC3425584

[20] Caldas AR Catita J Machado R Ribeiro A Cerqueira F Horta B. Omega-3-and resveratrol-loaded lipid nanosystems for potential use as topical formulations in autoimmune, inflammatory, and cancerous skin diseases. Pharmaceutics. (2021) 13:1202. 10.3390/pharmaceutics13081202 34452163 PMC8401194

[21] Kim J-H Park E-Y Ha H-K Jo C-M Lee W-J Lee SS. Resveratrol-loaded nanoparticles induce antioxidant activity against oxidative stress. Asian-Australas J Anim Sci. (2015) 29:288–98. 10.5713/ajas.15.0774 26732454 PMC4698710

[22] Figueroa-Robles A Antunes-Ricardo M Guajardo-Flores D. Encapsulation of phenolic compounds with liposomal improvement in the cosmetic industry. Int J Pharm. (2021) 593:120125. 10.1016/j.ijpharm.2020.120125 33253799

[23] Kirilenko V Gregoriadis G. Fat soluble vitamins in liposomes: studies on incorporation efficiency and bile salt induced vesicle disintegration. J Drug Target. (1993) 1:361–8. 10.3109/10611869308996095 8069579

[24] Maurer N Fenske DB Cullis PR. Developments in liposomal drug delivery systems. Expert Opin Biol Ther. (2001) 1:923–47. 10.1517/14712598.1.6.923 11728226

[25] Fahr A van Hoogevest P May S Bergstrand N Leigh ML. Transfer of lipophilic drugs between liposomal membranes and biological interfaces: consequences for drug delivery. Eur J Pharm Sci. (2005) 26:251–65. 10.1016/j.ejps.2005.05.012 16112849

[26] Padamwar MN Pokharkar VB. Development of vitamin loaded topical liposomal formulation using factorial design approach: drug deposition and stability. Int J Pharm. (2006) 320:37–44. 10.1016/j.ijpharm.2006.04.001 16707237

[27] Machado AR Pinheiro AC Vicente AA Souza-Soares LA Cerqueira MA. Liposomes loaded with phenolic extracts of Spirulina LEB-18: Physicochemical characterization and behavior under simulated gastrointestinal conditions. Food Res Int. (2019) 120:656–67. 10.1016/j.foodres.2018.11.023 31000284

[28] Caddeo C Teskač K Sinico C Kristl J. Effect of resveratrol incorporated in liposomes on proliferation and UV-B protection of cells. Int J Pharm. (2008) 363:183–91. 10.1016/j.ijpharm.2008.07.024 18718515

[29] Aviagen W. Ross 308: Broiler's Management Nutrition Specification. (2018). Available online at: eu.aviagen.com

[30] AOAC. (2012). Official Methods of Analysis of AOAC International, Association of Official Analytical Chemists. 19th Ed., Gaithersburg, MD: AOAC International.

[31] Mohan A Narayanan S Sethuraman S Krishnan UM. Novel resveratrol and 5-fluorouracil coencapsulated in PEGylated nanoliposomes improve chemotherapeutic efficacy of combination against head and neck squamous cell carcinoma. Biomed Res Int. (2014) 2014:424239. 10.1155/2014/424239 25114900 PMC4119704

[32] Al-Khalaifah HS Shahin S Omar AE Mohammed HA Mahmoud H Ibrahim D. Effects of graded levels of microbial fermented or enzymatically treated dried brewer's grains on growth, digestive and nutrient transporter genes expression and cost effectiveness in broiler chickens. BMC Vet Res. (2020) 16:1–15. 10.1186/s12917-020-02603-0 33153443 PMC7643478

[33] Farahat M Ibrahim D Kishawy A Abdallah H Hernandez-Santana A Attia G. Effect of cereal type and plant extract addition on the growth performance, intestinal morphology, caecal microflora, and gut barriers gene expression of broiler chickens. Animal. (2021) 15:100056. 10.1016/j.animal.2020.100056 33573933

[34] Ibrahim D Abdelfattah-Hassan A Arisha AH Abd El-Aziz RM Sherief WR Adil SH. Impact of feeding anaerobically fermented feed supplemented with acidifiers on its quality and growth performance, intestinal villi and enteric pathogens of mulard ducks. Livest Sci. (2020) 242:104299. 10.1016/j.livsci.2020.104299

[35] Ibrahim D Ismail TA Khalifa E El-Kader A Shaimaa A Mohamed DI. Supplementing garlic nanohydrogel optimized growth, gastrointestinal integrity and economics and ameliorated necrotic enteritis in broiler chickens using a clostridium perfringens challenge model. Animals. (2021) 11:2027. 10.3390/ani11072027 34359156 PMC8300316

[36] Livak KJ Schmittgen TD. Analysis of relative gene expression data using real-time quantitative PCR and the 2– ΔΔCT method. Methods. (2001) 25:402–8. 10.1006/meth.2001.1262 11846609

[37] Bancroft JD Gamble M. Theory and Practice of Histological Techniques. Amsterdam: Elsevier Health Sciences (2008).

[38] Mujahid A Yoshiki Y Akiba Y Toyomizu M. Superoxide radical production in chicken skeletal muscle induced by acute heat stress. Poult Sci. (2005) 84:307–14. 10.1093/ps/84.2.307 15742968

[39] Zhang C Zhao X Yang L Chen X Jiang R Jin S. Resveratrol alleviates heat stress-induced impairment of intestinal morphology, microflora, and barrier integrity in broilers. Poult Sci. (2017) 96:4325–32. 10.3382/ps/pex266 29053872

[40] Dong S Li H Gasco L Xiong Y Guo K Zoccarato I. Antioxidative activity of the polyphenols from the involucres of Castanea mollissima Blume and their mitigating effects on heat stress. Poult Sci. (2015) 94:1096–104. 10.3382/ps/pev101 25805834

[41] Ibrahim D Moustafa A Shahin S Sherief W Farag M Nassan M. Impact of fermented or enzymatically fermented dried olive pomace on growth, expression of digestive enzymes and glucose transporters genes, oxidative stability of frozen meat and economic efficiency of broiler chickens. Front Vet Sci. (2021) 8:442. 10.3389/fvets.2021.644325 34124216 PMC8193359

[42] He S Chen L He Y Chen F Ma Y Xiao D. Resveratrol alleviates heat stress-induced impairment of intestinal morphology, barrier integrity and inflammation in yellow-feather broilers. Anim Prod Sci. (2020) 60:1547–56. 10.1071/AN19218

[43] Azad M Kikusato M Maekawa T Shirakawa H Toyomizu M. Metabolic characteristics and oxidative damage to skeletal muscle in broiler chickens exposed to chronic heat stress. Comp Biochem Physiol A Mol Int Physiol. (2010) 155:401–6. 10.1016/j.cbpa.2009.12.011 20036750

[44] Mujahid A Pumford NR Bottje W Nakagawa K Miyazawa T Akiba Y. Mitochondrial oxidative damage in chicken skeletal muscle induced by acute heat stress. J Poult Sci. (2007) 44:439–45. 10.2141/jpsa.44.439

[45] Zhao R Shen GX. Functional modulation of antioxidant enzymes in vascular endothelial cells by glycated LDL. Atherosclerosis. (2005) 179:277–84. 10.1016/j.atherosclerosis.2004.11.013 15777542

[46] Zhang L Wang L Shi Z Wei X Chen J Zhao R. Expression of SGLT1 mRNA in duodenum, jejunum and ileum of weaning pigs and the effect of cysteamine on it. J Agri Biotech. (2006) 14:850–4.

[47] Circu ML Aw TY. Reactive oxygen species, cellular redox systems, and apoptosis. Free Radic Biol Med. (2010) 48:749–62. 10.1016/j.freeradbiomed.2009.12.022 20045723 PMC2823977

[48] Arulselvan P Subramanian SP. Beneficial effects of Murraya koenigii leaves on antioxidant defense system and ultra structural changes of pancreatic β-cells in experimental diabetes in rats. Chem Biol Interact. (2007) 165:155–64. 10.1016/j.cbi.2006.10.014 17188670

[49] Sgambato A Ardito R Faraglia B Boninsegna A Wolf FI Cittadini A. Resveratrol, a natural phenolic compound, inhibits cell proliferation and prevents oxidative DNA damage. Mut Res. (2001) 496:171–80. 10.1016/S1383-5718(01)00232-7 11551493

[50] Mohammed MA Abdulridha W Abd AN. Thickness effect on some physical properties of the Ag thin films prepared by thermal evaporation technique. J Glob Pharma Technol. (2018) 10:613–9.

[51] Kang KW Lee SJ Kim SG. Molecular mechanism of nrf2 activation by oxidative stress. Antioxid Redox Signal. (2005) 7:1664–73. 10.1089/ars.2005.7.1664 16356128

[52] Zhu Y Zhang Y-J Liu W-W Shi A-W Gu N. Salidroside suppresses HUVECs cell injury induced by oxidative stress through activating the Nrf2 signaling pathway. Molecules. (2016) 21:1033. 10.3390/molecules21081033 27517893 PMC6273208

[53] Wang J Jia R Gong H Celi P Zhuo Y Ding X. The effect of oxidative stress on the chicken ovary: involvement of microbiota and melatonin interventions. Antioxidants. (2021) 10:1422. 10.3390/antiox10091422 34573054 PMC8472688

[54] Vanaja K Wahl M Bukarica L Heinle H. Liposomes as carriers of the lipid soluble antioxidant resveratrol: evaluation of amelioration of oxidative stress by additional antioxidant vitamin. Life Sci. (2013) 93:917–23. 10.1016/j.lfs.2013.10.019 24177602

[55] Caddeo C Nacher A Vassallo A Armentano MF Pons R Fernàndez-Busquets X. Effect of quercetin and resveratrol co-incorporated in liposomes against inflammatory/oxidative response associated with skin cancer. Int J Pharm. (2016) 513:153–63. 10.1016/j.ijpharm.2016.09.014 27609664

[56] Meng T Deng J Xiao D Arowolo MA Liu C Chen L. Protective effects and potential mechanisms of dietary resveratrol supplementation on the spleen of broilers under heat stress. Front Nutr. (2022) 9:821272. 10.3389/fnut.2022.821272 35651504 PMC9150503

[57] Moura CS Lollo PCB Morato PN Amaya-Farfan J. Dietary nutrients and bioactive substances modulate heat shock protein (HSP) expression: a review. Nutrients. (2018) 10:683. 10.3390/nu10060683 29843396 PMC6024325

[58] Sahebkar A Mohammadi A Atabati A Rahiman S Tavallaie S Iranshahi M. Curcuminoids modulate pro-oxidant–antioxidant balance but not the immune response to heat shock protein 27 and oxidized LDL in obese individuals. Phytother Res. (2013) 27:1883–8. 10.1002/ptr.4952 23494802

[59] Yu J Bao E Yan J Lei L. Expression and localization of Hsps in the heart and blood vessel of heat-stressed broilers. Cell Stress Chaperones. (2008) 13:327–35. 10.1007/s12192-008-0031-7 18350374 PMC2673943

[60] Singh CK Chhabra G Ndiaye MA Garcia-Peterson LM Mack NJ Ahmad N. The role of sirtuins in antioxidant and redox signaling. Antioxid Redox Signal. (2018) 28:643–61. 10.1089/ars.2017.7290 28891317 PMC5824489

[61] Oberdoerffer P Michan S McVay M Mostoslavsky R Vann J Park S-K. SIRT1 redistribution on chromatin promotes genomic stability but alters gene expression during aging. Cell. (2008) 135:907–18. 10.1016/j.cell.2008.10.025 19041753 PMC2853975

[62] Cao C Lu S Kivlin R Wallin B Card E Bagdasarian A. SIRT1 confers protection against UVB-and H2O2-induced cell death via modulation of p53 and JNK in cultured skin keratinocytes. J Cell Mol Med. (2009) 13:3632–3643. 10.1111/j.1582-4934.2008.00453.x 18681908 PMC4516512

[63] Ido Y Duranton A Lan F Weikel KA Breton L Ruderman NB. Resveratrol prevents oxidative stress-induced senescence and proliferative dysfunction by activating the AMPK-FOXO3 cascade in cultured primary human keratinocytes. PLoS ONE. (2015) 10:e0115341. 10.1371/journal.pone.0115341 25647160 PMC4315597

[64] Ansari A Rahman MS Saha SK Saikot FK Deep A Kim KH. Function of the SIRT 3 mitochondrial deacetylase in cellular physiology, cancer, and neurodegenerative disease. Aging Cell. (2017) 16:4–16. 10.1111/acel.12538 27686535 PMC5242307

[65] Yu W Fu YC Zhou XH Chen CJ Wang X Lin RB. Effects of resveratrol on H2O2-induced apoptosis and expression of SIRTs in H9c2 cells. J Cell Biochem. (2009) 107:741–7. 10.1002/jcb.22169 19415680

[66] Ibrahim D El Sayed R Abdelfattah-Hassan A Morshedy A. Creatine or guanidinoacetic acid? Which is more effective at enhancing growth, tissue creatine stores, quality of meat, and genes controlling growth/myogenesis in Mulard ducks. J Appl Anim Res. (2019) 47:159–66. 10.1080/09712119.2019.1590205

[67] Li Y Li F Duan Y Guo Q Wang W Wen C. The protein and energy metabolic response of skeletal muscle to the low-protein diets in growing pigs. J Agric Food Chem. (2017) 65:8544–51. 10.1021/acs.jafc.7b02461 28915727

[68] Zuo J Xu M Abdullahi YA Ma L Zhang Z Feng D. Constant heat stress reduces skeletal muscle protein deposition in broilers. J Sci Food Agric. (2015) 95:429–36. 10.1002/jsfa.6749 24871527

[69] Li X Zhang M Feng J Zhou Y. Myostatin and related factors are involved in skeletal muscle protein breakdown in growing broilers exposed to constant heat stress. Animals. (2021) 11:1467. 10.3390/ani11051467 34065334 PMC8160752

[70] Rajasekaran NS Shelar SB Jones DP Hoidal JR. Reductive stress impairs myogenic differentiation. Redox Biol. (2020) 34:101492. 10.1016/j.redox.2020.101492 32361680 PMC7199008

[71] Sandiford SD Kennedy KA Xie X Pickering JG Li SS. Dual oxidase maturation factor 1 (DUOXA1) overexpression increases reactive oxygen species production and inhibits murine muscle satellite cell differentiation. Cell Commun Signal. (2014) 12:1–15. 10.1186/1478-811X-12-5 24410844 PMC3895674

[72] Ibrahim D Al-Khalaifah HS Abdelfattah-Hassan A Eldoumani H Khater SI Arisha AH. Promising role of growth hormone-boosting peptide in regulating the expression of muscle-specific genes and related MicroRNAs in broiler chickens. Animals. (2021) 11:1906. 10.3390/ani11071906 34206912 PMC8300367

[73] Lian D Chen M-M Wu H Deng S Hu X. The role of oxidative stress in skeletal muscle myogenesis and muscle disease. Antioxidants. (2022) 11:755. 10.3390/antiox11040755 35453440 PMC9026549

[74] Ding X Cai C Jia R Bai S Zeng Q Mao X. Dietary resveratrol improved production performance, egg quality, and intestinal health of laying hens under oxidative stress. Poult Sci. (2022) 101:101886. 10.1016/j.psj.2022.101886 35526444 PMC9092510

[75] Ibrahim D Eldemery F Metwally AS Abd-Allah EM Mohamed DT Ismail TA. Dietary eugenol nanoemulsion potentiated performance of broiler chickens: orchestration of digestive enzymes, intestinal barrier functions and cytokines related gene expression with a consequence of attenuating the severity of E. coli O78 infection. Front Vet Sci. (2022) 9:847580. 10.3389/fvets.2022.847580 35812892 PMC9260043

[76] Yang H Wang Y Liu M Liu X Jiao Y Jin S. Effects of dietary resveratrol supplementation on growth performance and anti-inflam matory ability in ducks (Anas platyrhynchos) through the Nrf2/HO-1 and TLR4/NF-κB signaling pathways. Animals. (2021) 11:3588. 34944363 10.3390/ani11123588 PMC8698092

[77] Emami NK Jung U Voy B Dridi S. Radical response: effects of heat stress-induced oxidative stress on lipid metabolism in the avian liver. Antioxidants. (2020) 10:35. 10.3390/antiox10010035 33396952 PMC7823512

[78] Tang L-P Liu Y-L Zhang J-X Ding K-N Lu M-H He Y-M. Heat stress in broilers of liver injury effects of heat stress on oxidative stress and autophagy in liver of broilers. Poult Sci. (2022) 101:102085. 10.1016/j.psj.2022.102085 36055022 PMC9445375

[79] Ding K-N Lu M-H Guo Y-N Liang S-S Mou R-W He Y-M. Resveratrol relieves chronic heat stress-induced liver oxidative damage in broilers by activating the Nrf2-Keap1 signaling pathway. Ecotoxicol Environ Saf. (2023) 249:114411. 10.1016/j.ecoenv.2022.114411 36525949

